# Continuous Postoperative Antibiotic Irrigation via Catheter System Following Immediate Breast Reconstruction

**Published:** 2015-11-13

**Authors:** John Paul Tutela, David P. Duncan, S. Sean Kelishadi, Saeed Chowdhry, Travis Boyd, Jarrod A. Little

**Affiliations:** University of Louisville Department of Plastic Surgery, Louisville, Ky

**Keywords:** infection, catheter system, continuous antibiotic irrigation, mastectomy, breast reconstruction

## Abstract

Breast reconstruction with implantable devices is now the most common type of technique utilized following mastectomy. Because infections are one of the most common complications for the procedure and currently no one method has been proven to stand above the rest, we designed and implemented a novel technique that employed 24 hours continuous triple-antibiotic irrigation via a catheter-based system. From August 2009 to March 2012, 79 patients underwent tissue expander-based reconstruction from a single plastic surgeon. Forty-five consecutive patients underwent breast reconstructive surgery with implant-based reconstruction alone; the remaining 34 patients underwent breast reconstructive surgery with tissue expansion and closed continuous postoperative antibiotic irrigation. Incidences of infection, seroma, hematoma, and premature explantation were recorded. Both the rate of premature explant (20% vs 2.9%; *P* = 0.037) and surgical site infections (22.2% vs 5.8%, *P* = 0.060) decreased. Twenty-four hour continuous antibiotic irrigation is a useful adjunct to tissue expander breast reconstruction.

Between 1998 and 2008, 178,603 total mastectomies were performed in the United States. A total of 51,410 were followed by immediate reconstructions, 27,589 of which were implant-based procedures.^1^ Increasing annually by an average of 6% over this time period, the use of implantable devices became the most common reconstruction technique in 2008 occurring in approximately 258 per 1000 mastectomies versus 120 per 1000 mastectomies for immediate autologous reconstructions.^1^ It is important to note that breast reconstruction following mastectomy using tissue expanders carries a significant risk of complication. With the literature reporting an incidence between 2.5% and 29%, infection is repeatedly one of the most common complications for the procedure.^1^^-^5 There have been several proposed solutions to overcome implant infection; however, due to the fact that the mechanism by which surgical site infections (SSIs) occur is unclear, currently no one method stands above the rest in reducing infection. Surgical site infection of a breast prosthetic averages a cost of $4091 per case owing to longer hospital stays and additional procedures.^6^^,^^7^ This figure does not take into account the many hidden costs such as lost productivity, a less than optimal esthetic result, and the inherent psychological and physical morbidity.^6^

Irrigation of the surgical site has been used in many settings to reduce the likelihood of complications. Inspired by the first descriptions of the use of continuous wound irrigation to prevent infection by Carrel and Dehelly,^8^ Svedman et al^9^ developed a sophisticated irrigation system that efficiently delivers continuous or intermittent irrigation throughout a wound. Kasdan introduced the use of a polyurethane catheter irrigation system to continuously flush the surgical site with cold lactated Ringer's during the immediate postoperative period following Dupytren's contracture release. This method demonstrated a reduction in postoperative hematoma, edema, and pain following the procedure.^10^ Although there are few studies that demonstrate any significant decrease in infection rates in the setting of breast surgery, Adams et al^11^ demonstrated lower rates of infection and capsular contracture in cosmetic and reconstructive procedures using triple-antibiotic irrigation before closing. To our knowledge, no publication addresses the role of catheter-based continuous triple-antibiotic irrigation in reducing complications following immediate breast reconstruction with tissue expanders; our retrospective analysis explores the role of this technique in breast reconstruction.

## METHODS

79 consecutive patients underwent tissue expander breast reconstruction performed by a single surgeon following modified radical mastectomy for the treatment of breast carcinoma from August 2009 through March 2012. All patients were analyzed for the rates of complications as well as the existing comorbid conditions of smoking, diabetes, radiation, and obesity. Those with allergies to any of the antibiotics or materials used in the irrigation system were excluded from the study. To reduce variability within the subject population, we also excluded patients who underwent reconstruction with AlloDerm.

The patient population was divided into 2 groups. The first group consisted of 45 patients who underwent tissue expander–based reconstructive breast surgery following mastectomy; this was designated the control group. The second group, designated the intervention group, consisted of 34 patients who underwent tissue expander–based breast reconstruction following mastectomy with closed continuous antibiotic irrigation in the immediate 24 hours postoperatively. Of the 45 patients in the control group, 10 were smokers, 3 were diabetic, 6 had previously received radiation to the breast tissue, and 3 were obese. Of the 34 patients in group 2, 15 were current smokers, 1 was a diabetic, 4 had received radiation to the breast tissue, and 12 were obese, as seen in Table 1. The incidence of smokers in our patient population is approximately double that of the national average.^12^

Obtaining exact incidences and causes of postoperative SSI can be exceedingly difficult due to its nature as a clinical diagnosis. Presenting features following breast prosthetic procedures such as fever, rapidly evolving pain, breast erythema, induration, and fluid around the prosthesis are classic although highly variable.^13^ For this reason, the Centers for Disease Control and Prevention (CDC) propose that infections of the breast must occur less than 90 days after the procedure; involve the deep soft tissue layers; and have at least one of the following to meet the criteria required for classification as a SSI: purulent drainage from the surgical site, evidence of abscess, or spontaneous dehiscence or intentional reopening of a deep incision that is either culture-positive or not cultured in combination to fever, localized pain, or tenderness.^14^

The factors recorded between the 2 groups were SSIs as defined by the CDC criteria, premature explant, hematoma, and seroma.

## OPERATIVE TECHNIQUE

Cefazolin was given intravenously at an interval less than 30 minutes before skin incision for mastectomy and again before beginning the reconstruction. For patients with weight less than 110 kg, 1 g of cefazolin was given; 2 g of cefazolin were given to patients with weight more than 110 kg. The house surgical oncologist draped the patient and performed all mastectomies. After the surgical oncologist finished, a new sterile field was created over the one used for tissue removal.

**Figure 1:**
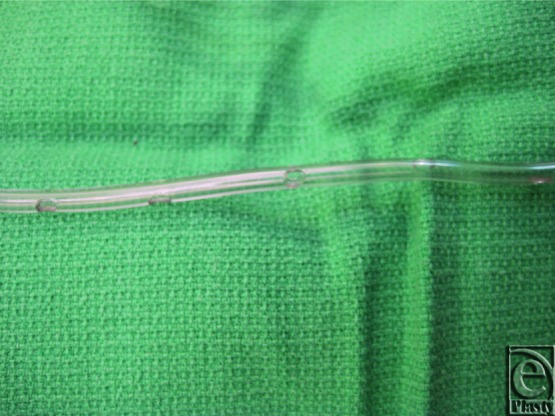
Close up of irrigation system with holes cut into sterile arterial line tubing.

**Figure 2:**
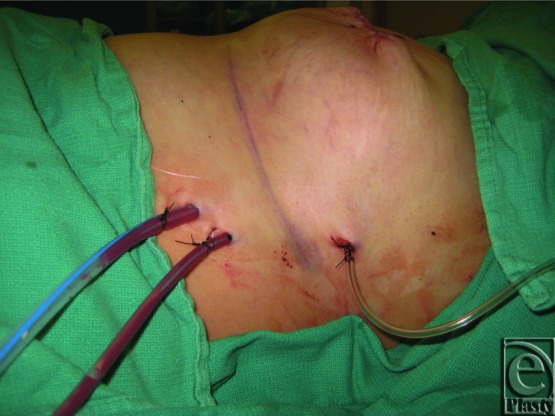
Irrigation tubing seen on the right (superior) of the photo graph, and two drains seen on the left (inferior) of the photographs.

**Figure 3:**
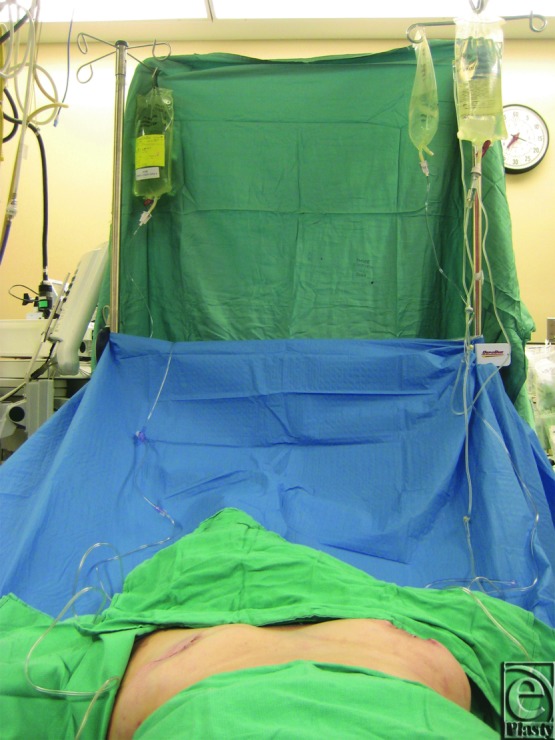
One liter bags of irrigation used for the irrigation system.

All of the reconstructive cases used a submuscular pocket and tissue expanders were placed using meticulous aseptic technique. No cases used acellular dermal matrix. The irrigation system is composed of sterile pressure tubing that is found in an arterial line extension kit. Small perforations were created on the distal end of the tubing as seen in Figure 1. The irrigation tubing is placed over the superior aspect of the tissue expander and under the pectoralis major muscle. One drain was placed inferiorly over the expander but under the muscle, and the other drain was placed over the superior aspect of the muscle. Figures 2 and 3 depict the entire irrigation system and how the patient will travel back to the floor, with an antibiotic irrigation solution consisting of a 1-L saline bag containing 80 mg of gentamicin, 1 g of cefazolin, and 50,000 units of bacitracin and a tubing set for each side. The irrigation is set to flow at a rate of 40 mL/hr and is left in place for 24 hours.

## RESULTS

The 2 treatment groups of breast reconstruction with tissue expander alone and breast reconstruction with closed continuous antibiotic irrigation were analyzed and compared. Both groups had an average age of 48 years. Of the 45 patients in group 1, who underwent breast reconstructive surgery with tissue expansions alone, 10 had SSIs (22.2%), 9 had premature explant (20%), zero developed a hematoma (0%), and 4 developed a seroma (8.8%). Of the 34 patients that underwent breast reconstructive surgery with tissue expansion and closed continuous postoperative antibiotic irrigation, 2 had SSIs (5.8%), 1 had premature explant (2.9%), 3 developed a hematoma (8.8%), and 3 developed a seroma (8.8%) (Table 2).

**Figure 4:**
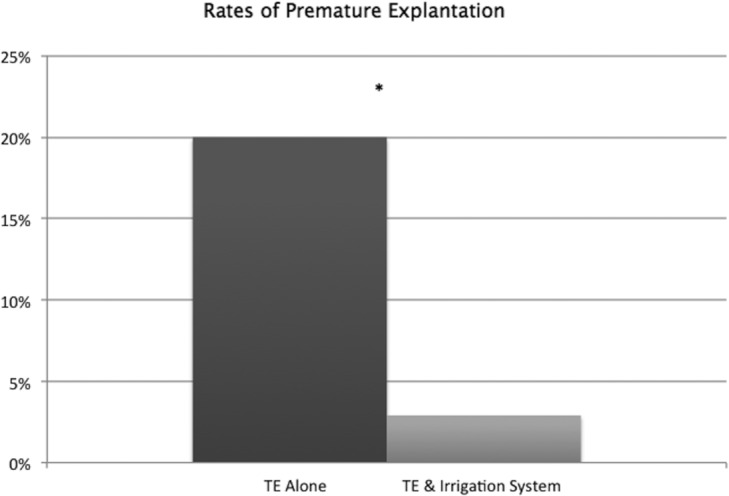
Rate of premature explantations.

**Figure 5:**
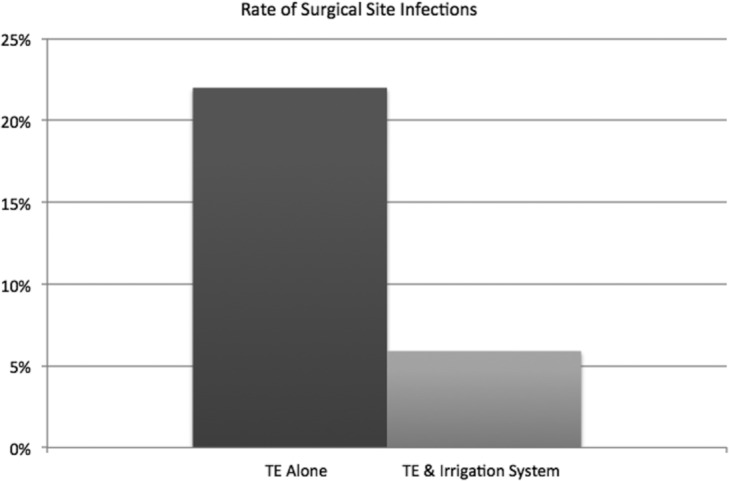
Rate of surgical site infections.

When closed continuous antibiotic irrigation was implemented in the immediate 24 hours postoperatively of tissue expander implantation, the incidence of premature explant showed a statistically significant decrease from 20% to 2.9% (*P* = .0373) (Fig 4). Occurrence of SSIs also had a downward trend from 22.2% to 5.8% (*P* = .060) (Fig 5).

## DISCUSSION

The goal in breast reconstruction is to provide the patient with an optimal cosmetic result while minimizing complications without interfering with the treatment of breast cancer. The implantation of tissue expanders immediately following mastectomy has become an increasingly popular technique in the attempt to accomplish these goals; however, using this procedure does not come without its pitfalls, one of which, infection, is particularly problematic.

Francis et al^15^ investigated independent risk factors for infection in tissue expander–based breast reconstruction and found that placement of a tissue expander within a breast that had previously had one removed was the greatest risk factor for infection, with an odds ratio of 4.94. These findings alone illustrate the necessity for the development of more effective methods of infection prevention. Our team noticed infections rates were at the upper end of the range reported in the literature and was determined to improve patient outcomes. Since the 2 most important determinants of infection are underlying patient condition and surgical technique,^2^^,^^3^^,^^7^^,^^11^^,^^15^ we felt we needed to alter our technique.

Before beginning the study, we had adhered to the suggestions laid out in the paper from Lane et al.^7^ The first of which is prevention of perioperative hypothermia, which has been associated with increased infection rates in both colorectal procedures,^16^ cholecystectomy,^17^ and presumptively “clean” procedures.^18^ Complete abstinence from hair removal was observed because of its association with increased SSI rates.^7^^,^^19^^,^^20^ The remaining suggestions to reduce SSI required long-term preventative measures such as smoking cessation, glycemic control, and weight loss.^7^ While the patients were counseled on the risks associated with each of these factors, our study did not seek to alter these factors. Instead, we recorded the prevalence at which they were present within the study population and found no statistical difference between groups.

In addition to the prevention of perioperative hypothermia, we implemented the following practices recommended by Adams et al^11^ before beginning our study^11^:

- avoiding blunt instrumentation and creating a pocket under direct vision;
- during pocket dissection, allow tissue expanders to soak in irrigation fluid;
- irrigation of pocket with irrigation solution without any active evacuation;
- cleansing of skin surrounding the incisions with irrigation solution;
- dawning new gloves after pocket dissection before expander handling;
- aseptic implant insertion;
- minimizing implant manipulation after insertion; and
- washing gloves occasionally in antibiotic solution if excessive manipulation is required.

Despite this, we felt our infection rate was unacceptable and in need of improvement.

The literature reports the implementation of catheter-based continuous irrigation for multiple purposes including the salvage in infected cartilage,^4^ the reduction of hematoma in Dupuytren's contracture release,^17^ the improvement of peritoneal carcinomatosis utilizing hyperthermic intraperitoneal chemotherapy,^21^^-^23 and a shorter time interval to primary closure following damage control surgery as a result of trauma.^23^ While these studies are different in their primary goals and outcomes, conceptually they all demonstrate the utility of continuous irrigation as an adjunct therapeutic modality to reduce of complications. Knowing that one-time irrigation of the surgical site with antibiotic solution decreased overall infection rates^24^^-^29 and that the use of continuous irrigation in the circumstances listed above provided measurable benefit, we decided to institute a protocol of continuous antibiotic irrigation into our expander pockets.

Referring to previous studies that showed that the breast is colonized with many types of bacteria, the complete list of which can be found in Table 3,^30^^,^^31^ we selected antibiotics that would cover the organisms observed while being cognizant of those antibiotics that were readily available and of reasonable cost. Of particular concern is the patient who cannot tolerate these antibiotics due to systemic allergic reactions. For this reason, patients with prior history or documentation of an adverse reaction to any antibiotic were excluded from the study.

Complications following mastectomy alone are reportedly as high at 48%; however, many of these studies follow patients for multiple years after surgical intervention because a significant proportion of breast implant infections arise indolently, often leading to equivocal clinical signs and variable clinician diagnoses even with the implementation of CDC diagnostic criteria.^5^^,^^32^^-^35 Focusing more on the perioperative and subacute complications following mastectomy potentially could have introduced bias toward calculating a lower incidence of complications. For this reason, we determined that permanent implant removal was the most objective and primary end point of our study.

When analyzing the data, we found a significant decrease of premature explantation of tissue expanders in implant-based breast reconstruction when using a closed continuous antibiotic irrigation system.

We feel that the irrigation offers 2 mechanisms for decreasing infection. The first is the actual antibiotic properties of the solution itself, and the second is the physical removal of debris from the mastectomy pocket. By continual irrigation of the debris from the pocket, small amounts of tissue and blood are washed away and with it decreasing the nidus to infection. More studies would be needed to prove this as the mechanism.

Limitations of this study are its heterogeneous sample population, small sample size, nonrandomization, and a control group infection rate on the upper end of normal. More studies are needed to evaluate the role of continuous antibiotic irrigation in reducing infection and removal rates of permanent implants that occur following mastectomy with or without immediate reconstruction with tissue expanders; however, we found that continuous catheter-based antibiotic irrigation reduces the rate of premature removal of the implants in expander-based breast reconstruction in our patient population.

## Figures and Tables

**Table 1:** Number of comorbid conditions by group

	Tobacco abuse	Diabetes	Obesity	Radiation therapy
TE alone (n = 45)	10 (22.2%)	3 (6.6%)	3 (6.6%)	6 (13.3%)
TE and irrigation system (n = 34)	15 (44.1%)	1 (2.9%)	12 (35.3%)	4 (11.8%)

TE indicates tissue expansion.

**Table 2:** Clinical variables in patients having undergone breast reconstructive surgery

	Total surgical site infections (rate)	Total premature explants (rate)	Total hematomas (rate)	Total seroma (rate)
Tissue expansion (n = 45)	10 (22%)	9 (20%)	0 (0.0)	4 (8.8%)
Tissue expansion and irrigation system (n = 34)	2 (5.9%)	1 (2.9%)	3 (8.8%)	3 (8.8%)

**Table 3:** Microorganisms identified in specimens obtained from women undergoing breast augmentation or reduction^31^

Aerobic
Coagulase-negative staphylococci (53%)
Diphtheroids (9%)
Lactobacilli (9%)
Bacillus spp. (5%)
β-hemolytic streptococci (3%)
Anaerobic
Propionibacterium acnes
